# Health worker performance in the management of paediatric fevers following in-service training and exposure to job aids in Kenya

**DOI:** 10.1186/1475-2875-9-261

**Published:** 2010-09-18

**Authors:** Beatrice Wasunna, Dejan Zurovac, Jane Bruce, Caroline Jones, Jayne Webster, Robert W Snow

**Affiliations:** 1 Malaria Public Health and Epidemiology Group, KEMRI/Wellcome Trust Research Programme, Centre for Geographic Medicine Research-Coast (CGMRC), P.O. Box 43640-00100 GPO, Nairobi, Kenya Nairobi, Kenya; 2 London School of Hygiene and Tropical Medicine (LSHTM), Disease Control and Vector Biology Unit (DCVBU), Keppel Street, WCIE 7HT, London, UK; 3 Centre for Tropical Medicine, Nuffield Department of Clinical Medicine, University of Oxford, CCVTM, Oxford, UK; 4 Center for International Health and Development, Boston University School of Public Health, 85 East Concord Street, 5th Floor, Boston, MA 02118, USA; 5 KEMRI/Wellcome Trust Research Programme, Centre for Geographic Medicine Research-Coast (CGMRC), P.O. Box 230-80108, Kilifi, Kenya

## Abstract

**Background:**

Improving the way artemether-lumefantrine (AL) is provided to patients attending clinics is critical to maximize the benefit of this new medicine. In 2007, a new initiative was launched in one part of Kenya to improve malaria case-management through enhanced in-service training and provision of job aids.

**Methods:**

An evaluation of the intervention using pre- and post-intervention cross sectional health facility surveys was conducted in Bondo district. The surveys included: audit of government health facilities, health worker structured interviews and exit interviews with caretakers of sick children below five years of age. The outcome indicators were the proportions of febrile children who had AL prescribed, AL dispensed, and four different dispensing and counseling tasks performed.

**Results:**

At baseline 33 government health facilities, 48 health workers and 386 febrile child consultations were evaluated. At follow-up the same health facilities were surveyed and 36 health workers and 390 febrile child consultations evaluated. The findings show: 1) no health facility or health worker was exposed to all components of the intervention; 2) the proportion of health workers who received the enhanced in-service training was 67%; 3) the proportion of febrile children with uncomplicated malaria treated with the first-line anti-malarial drug, artemether-lumefantrine (AL), at health facilities where AL was in stock increased from 76.9% (95%CI: 69.4, 83.1) to 87.6% (95% CI: 82.5, 91.5); 4) there were modest but non-significant improvements in dispensing and counseling practices; and 5) when the analyses were restricted to health workers who received the enhanced in-service training and/or had received new guidelines and job aids, no significant improvements in reported case-management tasks were observed compared to baseline.

**Conclusion:**

In-service training and provision of job aids alone may not be adequate to improve the prescribing, dispensing and counseling tasks necessary to change malaria case-management practices and the inclusion of supervision and post-training follow-up should be considered in future clinical practice change initiatives.

## Introduction

By 2009, every country in Africa had transitioned to new, effective artemisinin-based combination therapy (ACT) to manage uncomplicated malaria. Despite efforts to expand accessibility to ACT through private and retail sectors, most ACT drugs are only prescribed by health workers who work within the formal government and mission sector. Ensuring that the new ACT is prescribed according to national guidelines with the provision of adequate dispensing and counseling is critical to maximize patients' adherence [1,2] and treatment successes [3].

Non-adherence to malaria case management guidelines has been commonly reported in the past under ACT and non-ACT policies [4-7]. Complex multifaceted interventions containing high-quality educational strategies, intensive supervision, group processes, performance monitoring using audit and feedback and quality improvement schemes has been suggested as interventions most likely to improve adherence to guidelines in low income countries [8-10]. However, there is limited evidence on whether packages of interventions that do not include on-going interventions such as supportive supervision have an impact on health workers prescribing, dispensing and counseling practices. Here a pre-post-evaluation of such a package that aimed to improve how ACT is administered to febrile children presenting to government clinics in a highly malaria endemic area of Kenya is reported.

## Methods

### Intervention

#### Overview

A combined community and health facility intervention was launched with the aim of addressing the gaps in access to prompt and effective treatment with artemether-lumefantrine (AL), Kenya's recommended first-line treatment for uncomplicated malaria. The intervention was developed and implemented by Population Services International (PSI/Kenya) and the Division of Malaria Control (DOMC), Ministry of Public Health and Sanitation and funded by Pfizer under their Mobilize against Malaria Initiative [11]. The focus of delivery was limited to Nyanza Province, the most malaria endemic region of Kenya located around Lake Victoria with approximately 184 government health facilities serving a population of 5.56 million people in 2009. The intervention comprised two key strategies a) increasing awareness among the community of the need that children with fever attend clinics promptly, under the banner '*haraka upesi*' the Kiswahili meaning of prompt action; and b) improving the performance of health workers at facilities, focusing on reinforcement of AL treatment for febrile children and adherence to recommended dispensing and counseling tasks in accordance with national guidelines. This paper considers the second component of this strategy.

#### Malaria case-management training

Enhanced in-service health worker case-management training sessions were conducted in February 2009 and August 2009. Cadres of health staff involved in the training included: nurses, laboratory technicians, public health officers, clinical officers and doctors. The training sessions took place over three days in the form of workshops sited outside of the health workers' working stations. It consisted of several units including: the epidemiology of malaria in Kenya, diagnosis-treatment-counseling-drug dispensing for uncomplicated malaria, diagnosis and treatment of severe malaria, prevention and management of malaria in pregnancy, basic techniques related to stock management of AL, principles of monitoring and evaluation and practical sessions on diagnostic procedures using blood slides and rapid diagnostic tests [RDTs]. One day of the training was devoted to the management of uncomplicated malaria. No follow up of the training was provided.

The training was facilitated by the national DOMC trainers of trainers (TOTs) who are specialists in the different training units and have previously undertaken case-management training of health workers. The training format and teaching modalities included lectures, group discussions, case scenarios, role plays and question and answers quizzes. Between October and November 2008, a two-day pilot of the training with health workers and national trainers was undertaken in Kisumu East and Kisumu West districts in Nyanza province to establish the duration and feasibility of the intervention training. The feedback given by the health workers from this pilot was that overall duration of the training required more than two days to cover successfully all training units. Following this feedback the DOMC and PSI concluded that three-day training would provide adequate time to cover all training programme according to the curriculum. Furthermore, considering that peripheral health facilities often have only one health worker any training duration beyond three days was deemed unacceptable since it would have resulted in substantial disruption of the service delivery.

#### Training materials

The training curriculum, facilitators' manual on the diagnosis, treatment and prevention of malaria and job aids were developed by PSI/Kenya in collaboration with the Ministry of Public Health and Sanitation's, Division of Malaria Control (DOMC), case management working group partners and educational specialists. The training materials covered all of the training topics including those where deficiencies in practices were detected in the past and those which were the focus of the '*haraka upesi*' intervention - prompt AL treatment for febrile children and health workers' adherence to recommended case management. The manuals included objectives, content and modalities of delivery for each training unit. Alongside the training, the job aids were piloted to assess health workers understanding of the job aids messages. The key recommendation from the pilot, that was included in the final intervention package, was that for ease-of-use health workers requested a booklet encompassing miniature wall charts. Therefore, of relevance for the evaluation of the management of febrile children below 5 years of age, the following training materials were distributed to each health worker who attended the training: 1) revised national guidelines for the diagnosis, treatment and prevention of malaria for health workers; 2) participant's training manual based on revised national guidelines; and 3) five job aids promoting presumptive AL treatment of febrile children and dispensing and counseling tasks for patients with AL prescribed. Of five job aids disseminated, three were in wall chart or poster format, one was a booklet with miniature versions of the wall charts and one was the folder for the training materials with the '*haraka upesi*' banner.

The target of the training was universal coverage of all front-line health workers providing outpatient services. By the end of August 2009 an estimated 962 health workers had received the enhanced in-service training in 25 separate sessions at different locations across twelve districts within Nyanza province.

### Evaluation

#### Selection of study area

Bondo district was selected as the evaluation district from the twelve districts located in Nyanza Province. Bondo is on the shores of Lake Victoria where residents experience intense, perennial malaria transmission [12] and where previous studies have suggested that access to prompt treatment with AL was poor following the change in drug policy [13] and performance of health workers in the government sector sub-optimal with respect to managing febrile children [14,5]. The district has four main administrative divisions namely: Bondo, Usigu, Madiany and Rarieda. Thirty-six government health facilities, including three facilities that were established during the course of the study and not included here, serve the district population of over 280,000 inhabitants and staffed by 106 health workers. Two district hospitals provide in-patient care. Ninety-five health workers from Bondo District received the enhanced in-service training in February and August 2009 and were trained at a hotel in the district during four group sessions of between 21-33 health workers each.

#### Evaluation design

The design adopted for this evaluation was a pre-post intervention comparison of health worker case-management practices for febrile children aged less than five years. The key measured indicators reflected national guidelines, in-service training and job aid recommendations and included the following: 1) proportion of febrile children having AL prescribed; 2) proportion of febrile children having AL prescribed and dispensed; 3) proportion of febrile children with AL prescribed and dispensed who had a) weight measured; b) first dose administered at the facility; c) advice provided on dosing schedule; and d) advice provided on what to do in case of vomiting.

#### Data collection

Cross-sectional health facility surveys were undertaken at all government health facilities in Bondo district between August and October 2008 (pre-intervention) and November to December 2009 (post-intervention). Data were collected using three main tools: a) health facility audit to assess drug stocks, availability of equipment and training materials; b) health worker structured interview to obtain information on health worker's demographics and exposure to training and guidelines; and c) exit interviews with caregivers of febrile children below five years of age presenting to the outpatient department on the survey day. All health workers attending the febrile children on the survey day were interviewed. All caregivers of children under five years with fever or history of fever who were not referred for hospitalization were interviewed on leaving the health facility. Data were collected at each facility over two consecutive survey days by seven survey teams, each composed of two nurses. Informed written consent was obtained from all interviewed health workers and caretakers of sick children. Training and concordance testing were undertaken over a five day period immediately prior to data collection. Concordance testing for exit and health worker interviews was assessed through role playing at health facilities not included in the survey until 96% concordance was achieved.

#### Data analysis

Data were doubled entered by four independent data entry clerks using Microsoft Access customized software (Microsoft Inc, Redmond, Washington). Data files were subjected to a verification programme to check for data entry errors and these were corrected by referring to the original questionnaires. All analyses were performed using STATA, version 11 (StataCorp, College Station, Texas).

The primary pre- and post-intervention analysis included febrile children weighing five kilograms and above presenting for an initial visit at health facilities where AL was in stock on the survey day. The children with negative test result were excluded from the analyses. This analysis reflected effectiveness of the intervention regardless of the exposure to the intervention. However, due to incomplete coverage of health workers with all intervention components, two post-intervention analyses were additionally performed. The first analysis was restricted to febrile children seen by health workers who received the enhanced training, and the second analysis included children seen by health workers who in addition to the enhanced training had either access to guidelines or the participants' training manual, and at least one of the five job aids promoting the same case management recommendations in different formats. The latter analysis was defined as the minimum intervention package. Since none of the children were seen by health workers who were exposed to all intervention components the analysis reflecting the full intervention package could not be performed. Data are presented as percentages, with corresponding 95% confidence intervals (CIs) adjusted for clustering at health facility level.

### Ethical considerations

Ethical approval was obtained from the KEMRI/National ethical review committee (KEMRI SSC number 1375) and the London School of Hygiene and Tropical Medicine Ethics Committee (Ethical approval number: 5313)

## Results

### Description of the study sample and intervention coverage

During the pre-intervention survey, conducted between August and October 2008, 33 health facilities, 48 health workers and 599 consultations for febrile children were assessed. After the training sessions, in November 2009, 36 health workers working at the same health facilities and 541 consultations were assessed. No health worker or caretakers refused to participate in the study. The majority of assessed facilities were dispensaries (75.8%) followed by health centres (18.2%) and hospitals (6.1%). During both, pre-intervention and post-intervention surveys, nurses represented the majority of health workers (87.5% and 72.2% respectively), followed by clinical officers (12.5% and 17.0% respectively). Of the 33 health facilities, only one facility during the pre-intervention survey did not have any AL pack in stock and observations from this facility were excluded from the patient level analysis. Paediatric AL formulations were widely available during both surveys: between 94-100% of facilities stocked AL six and AL twelve pack formulations. Malaria diagnostic services were provided at 24.2% and 27.3% of facilities during the pre- and post-intervention survey, respectively.

Health workers and health facility coverage with the components of the intervention as assessed during the post-intervention survey are presented in Table 1. Of 36 interviewed health workers who performed outpatient consultations for febrile children on survey days, 67% had received the intervention training and 61% had access to either a personal or facility copy of the national malaria case management guidelines. At the 33 facilities, the most commonly observed job-aid was a wall chart with an algorithm for assessing and treating children below five years (76%), followed by the poster recommending presumptive treatment of childhood fevers with AL (58%) and AL dispensing wall chart (52%). Less than half of the facilities had a participant's training manual (46%), booklets with miniature wall charts (42%), and only 12% had folders with the '*haraka upesi*' banner. None of the health workers had received all components of the intervention and only 50% (18/36) were exposed to the minimum intervention package as defined above.

**Table 1:** Health worker and health facility intervention components

Health worker measured components (N = 36)	N	%
Health worker received PSI in-service training	24	67.0
Health worker has access to national malaria guideline	22	61.1

**Health facility measured components (N = 33)**		

AL dispensing wall chart exposed	17	51.5
Algorithm for assessing and treating children < 5 years exposed	25	76.0
Poster recommending presumptive treatment of childhood fevers with AL exposed	19	58.0
Booklet with miniature case management wall charts available	14	42.4
Folder recommending presumptive treatment of childhood fevers with AL available	4	12.1
Participants' training manual available	15	45.5

**Health worker's exposure to the intervention package (N = 36)**		

HW exposed to full intervention package including all components	0	0
HW exposed to minimum intervention package defined as PSI trained, having access to guidelines or training manual, and at least one of five remaining job aids	18	50.0

### Anti-malarial treatment practices for febrile children < 5 years of age in 2008 and 2009

Of 599 consultations assessed for febrile children during the pre-intervention survey in 2008, 386 children met the inclusion criteria for the case management analysis. The remaining 213 children were excluded from the analysis because they attended the facility for a follow-up visit (156), weighed less than five kilograms (4), had negative routine malaria slide result (14) or were seen at a facility without any AL in stock (39). During the post-intervention survey in 2009, of 541 consultations assessed 390 children met the inclusion criteria while the remaining 151 children were follow up visit (118), weighed less than five kilograms (23) or had negative routine malaria slide result (23). Finally, the restricted post-intervention analyses included 249 children seen by health workers who had received the enhanced in-service training and 174 seen by health workers who received the minimum intervention package.

Case management practices for febrile children before and after the intervention are presented in Table 2. The proportion of febrile children having AL prescribed was relatively high during the pre-intervention survey, 76.9% (95% CI: 69.4-83.1), yet it increased to 87.6% (95% CI: 82.5-91.5) during the post-intervention survey. Despite the availability of AL at all health facilities included in the analysis, 9.7% of children who were prescribed AL during the pre-intervention survey did not have AL dispensed before leaving the facility, thus resulting in only 69.4% (95% CI: 60.2-77.4) of children known to have AL prescribed and dispensed. There was an eighteen percentage point increase in the numbers of children leaving the health facility with recommended AL during the post-intervention survey and none of the children who did not have AL prescribed left facility with AL dispensed.

**Table 2:** Case-management practices for febrile children - comparison between pre- and post-intervention

	2008			2009								
	**Pre-intervention**			**Post-intervention**			**Restricted to children seen by trained HWs**			Restricted to children seen by HWs exposed to minimum package of intervention*		

	**N**	**%**	**95% CI**	**N**	**%**	**95% CI**	**N**	**%**	**95% CI**	**N**	**%**	**95% CI**

**Febrile children**	**N = 386**			**N = 390**			**N = 249**			**N = 174**		

AL prescribed	297	76.9	69.4, 83.1	342	87.6	82.5, 91.5	213	85.5	79.2, 90.2	148	85.1	75.9, 91.1
Other anti-malarial prescribed	33	8.5	5.5, 13.0	10	2.6	1.3, 5.1	8	3.2	1.5, 7.0	7	4.0	1.7, 9.0
AL prescribed and dispensed	268	69.4	60.2, 77.4	342	87.6	82.5, 91.5	213	85.5	79.2, 90.2	148	85.1	75.9, 91.1

**Febrile children with AL dispensed**	**N = 268**			**N = 342**			**N = 213**			**N = 148**		

Weight measured	199	74.2	58.1, 85.7	270	79.0	64.8, 88.4	157	73.7	54.6, 86.7	113	76.3	53.0, 90.3
First dose given at the health facility	58	21.6	10.4, 40.0	115	33.6	20.0, 50.6	62	29.1	14.3, 50.2	56	37.8	18.5, 62.0
Dosage to take at home explained	264	98.5	95.2, 99.5	339	99.1	97.4, 99.7	211	99.1	96.5, 100	147	99.3	94.5, 99.9
Advise on vomiting provided	9	3.3	1.6, 6.7	27	8.0	3.3, 17.6	11	5.1	2.4, 10.6	11	7.4	3.6, 14.5
At least three of the four counseling and dispensing tasks provided	54	20.1	10.2, 35.9	119	34.8	21.0, 52.1	62	29.1	14.4, 50.1	56	37.8	19.0, 61.7

*Minimum intervention package includes: exposure to training plus access to guidelines or training manual plus at least one of five job aids

During both surveys, nearly all (98.5% and 99.1% respectively) caretakers of children for whom AL was dispensed, reported that the health workers provided advice on completion of the AL treatment regimen. Small post-intervention increases were observed in comparison to the pre-intervention survey in the proportion of children having AL dispensed who were weighed, who were administered the first AL dose at the health facility and who were advised what to do in case of vomiting; however none of these differences were statistically significant (Table 2). During both surveys few children (1.5% before and 5.0% after the intervention) received all four AL dispensing and counseling tasks while the performance of at least three out of four tasks increased from 20.1% (95% CI: 10.2-35.9) to 34.8% (95% CI: 21.0-52.1) although the difference was not statistically significant.

Despite improvements in some indicators post intervention, the analyses restricted to children seen by health workers with enhanced in-service training and those seen by health workers with access to enhanced training, guidelines and job aids, showed no statistically significant differences in any indicator compared to the pre-intervention analyses (Table 2).

## Discussion

The MAM initiative was launched to improve coverage of trained health workers and case management practices in Nyanza Province, an area harbouring the highest malaria endemicity in Kenya, but characterized with low access to ACT [13] and reporting suboptimal coverage and case management practices during the early evaluation of ACT implementation in 2006 [5]. An optimistic target was set at 100% of health workers trained and exposed to intervention job aids. Our evaluation revealed that in the government health facilities in Bondo district about two-thirds (67%) of health workers had received the enhanced in-service training, but only 50% had received both the training and had some access to guidelines and job aids. None of the health workers had been exposed to the full package of the intervention as initially planned. This suboptimal coverage contributed to poor performance based on several measured indicators and precluded a proof-of concept evaluation that in-service-training and provision of supporting case-management materials would improve the way children are managed for malaria by 25 percentage points.

Where parts of the intervention had reached health workers and their facilities, there were modest improvements in indicators that were linked to the intervention banner '*haraka upesi*' notably AL prescription for febrile children increased by 10% post-intervention (Table 2). This could not however, be directly attributed to the intervention training. There was an increase, albeit non-significant, in the proportion of children who had the first dose of ACT administered at the health facility (Table 2). Administration of the first dose in a health facility has been associated with more successful treatment outcomes. A study undertaken in Tanzania reported that caregivers of febrile children were more likely to adhere to treatment with an ACT if the first dose had been given at the health facility [2]. The provision of counseling on what do if a febrile child vomited AL was also shown in Tanzania to be associated with better adherence to ACT [15]. In this study this task was very rarely performed in either the pre- or post-intervention surveys, an observation reported in other settings [6].

Malaria case-management training was the key component of the intervention evaluated in Bondo. The training programme in the study area was based on fairly well developed curriculum and implemented to the best of our knowledge according to the planned format facilitated by experienced trainers from the national malaria control programme. Furthermore, indicators measured in this evaluation were relatively basic and change in practices following training could have been expected. Yet, when the analysis was restricted to health workers exposed to the intervention training there was no significant change in any of the outcome indicators. Several health worker performance studies have indicated that single one-off interventions without follow up often do not translate into the desired outcomes [16], notably single one off didactic training of health workers does not translate to improved case-management practices [10].

The intervention in Nyanza province made a special effort to combine training with provision of supporting job aids and wall charts. However, even when the analysis was restricted to health workers who had received the intervention training and had access to some of the support materials no additional improvements were observed compared to the pre-intervention period. The one important case-management intervention not included within the intervention package was on-going supervision and follow-up after the in-service training. This additional component may have enhanced single training or improved use of job aids, wall charts and guidelines.

There are several limitations of this study. Not all prescribing, dispensing and counseling tasks recommended in the national malaria treatment guidelines were evaluated in the present report because questions were unfortunately asked differently during pre and post intervention surveys. These include: dosing and advice on second dose after eight hours, administration after the meal and emphasis on full course completion. This limited our ability to examine the range of possible behavioural changes that were contingent on training. However, those reported reflect important features of good clinical practice and are likely to represent reasonable indicators of training and job aide impact. The study was undertaken in only one district of twelve where the intervention was implemented. However those implementing the in-service training and distribution of job aids did know which district would be evaluated and made additional efforts to increase intervention coverage in this district relative to other districts.

## Conclusion

Even enhanced, well funded in-service training and job aid development packages aimed at improving the way febrile patients are managed in health facilities does not reach all those who provide care within government health sectors. Improving intervention coverage must be a pre-requisite to any significant changes in the proportions of patients managed effectively with new ACT medicines. However, where these interventions reached health workers and facilities only modest improvements were observed in the prescription and dispensing of AL and counseling provided to caretakers. Despite limitations of the present study we would urge caution in future large scale investments in in-service training and provision of job aids without other features of support, such as supervision, to enhance the value of training and printed materials. Finally, it is also important that the quality of the training is optimized to ensure effective content delivery in achieving desired case-management practices.

## Competing interests

Pfizer Inc. provided funding for this study but were not involved in study design, data collection, and interpretation of the results or decisions to submit the final manuscript. DZ and RWS have participated in the Novartis National Malaria Control Programme Best Practice Workshops in Africa and have received consultative fees for their participation.

## Authors' contributions

BW was responsible for study design, data cleaning, analysis, interpretation and writing of the final manuscript. DZ provided design and analysis support. JB provided design and statistical support. JW and CJ provided guidance on survey design and interpretation of the results. RWS was responsible for overall intellectual direction, study interpretation and contributed to the preparation of the final manuscript. All authors have read and approved the final manuscript.
